# Transforming growth factor-β1 induces matrix metalloproteinase-9 and cell migration in astrocytes: roles of ROS-dependent ERK- and JNK-NF-κB pathways

**DOI:** 10.1186/1742-2094-7-88

**Published:** 2010-12-06

**Authors:** Hsi-Lung Hsieh, Hui-Hsin Wang, Wen-Bin Wu, Po-Ju Chu, Chuen-Mao Yang

**Affiliations:** 1 Department of Nursing, Division of Basic Medical Sciences, Chang Gung Institute of Technology, Tao-Yuan, Taiwan; 2 Department of Pharmacology, Chang Gung University, Tao-Yuan, Taiwan; 3 Department of Medicine, Fu Jen Catholic University, Hsin-Chuang, Taipei County, Taiwan; 4 Department of Biomedical Sciences, Chang Gung University, Tao-Yuan, Taiwan

## Abstract

**Background:**

Transforming growth factor-β (TGF-β) and matrix metalloproteinases (MMPs) are the multifunctional factors during diverse physiological and pathological processes including development, wound healing, proliferation, and cancer metastasis. Both TGF-β and MMPs have been shown to play crucial roles in brain pathological changes. Thus, we investigated the molecular mechanisms underlying TGF-β1-induced MMP-9 expression in brain astrocytes.

**Methods:**

Rat brain astrocytes (RBA-1) were used. MMP-9 expression was analyzed by gelatin zymography and RT-PCR. The involvement of signaling molecules including MAPKs and NF-κB in the responses was investigated using pharmacological inhibitors and dominant negative mutants, determined by western blot and gene promoter assay. The functional activity of MMP-9 was evaluated by cell migration assay.

**Results:**

Here we report that TGF-β1 induces MMP-9 expression and enzymatic activity via a TGF-β receptor-activated reactive oxygen species (ROS)-dependent signaling pathway. ROS production leads to activation of extracellular signal-regulated kinase 1/2 (ERK1/2) and c-Jun-N-terminal kinase (JNK) and then activation of the NF-κB transcription factor. Activated NF-κB turns on transcription of the MMP-9 gene. The rat MMP-9 promoter, containing a NF-κB *cis*-binding site, was identified as a crucial domain linking to TGF-β1 action.

**Conclusions:**

Collectively, in RBA-1 cells, activation of ERK1/2- and JNK-NF-κB cascades by a ROS-dependent manner is essential for MMP-9 up-regulation/activation and cell migration induced by TGF-β1. These findings indicate a new regulatory pathway of TGF-β1 in regulating expression of MMP-9 in brain astrocytes, which is involved in physiological and pathological tissue remodeling of central nervous system.

## Background

Matrix metalloproteinases (MMPs) are a large family of zinc-dependent endopeptidases that play an important role in the turnover of extracellular matrix (ECM) and function in physiological and pathological processes [1]. In the central nervous system (CNS), MMPs, and MMP-9 especially, are implicated in development, morphogenesis, wounding healing, neurite outgrowth, and immune cell migration [2]. In addition, they also participate in the pathogenesis of several CNS diseases such as stroke, Alzheimer's disease, neuroinflammation, and malignant glioma [3]. Among members of the MMP family, MMP-9 has been shown to be elevated in various brain disorders [4-6]. Moreover, several pro-inflammatory mediators such as interleukin-1β (IL-1β), lipopolysaccharide, bradykinin (BK), and oxidized low-density lipoprotein (oxLDL) can induce MMP-9 expression and activity in cultured rat astrocytes [7-10], indicating that the expression and activation of MMP-9 may be regulated during brain injuries and inflammation.

Transforming growth factor-β (TGF-β) is a multifunctional cytokine that regulates a broad diversity of physiological and pathological processes, including tissue wound healing, inflammation, cell proliferation, differentiation, migration, and extracellualr matrix (ECM) synthesis [11-13]. Accordingly, TGF-β family members play an important role in early embryogenesis and in the homeostasis of adult tissues. However, several lines of evidence show that lack of coordination of TGF-β-dependent signaling often leads to a number of human diseases, including fibrosis [14,15], cancer [16,17], and autoimmune diseases [18]. Moreover, TGF-β is a key immune system modulator, TGF-β1 especially, that may have both pro- and anti-inflammatory effects in immune system depending on the cell type (11-13). Within the CNS, all three isoforms of TGF-βs family, *i.e*. TGF-β1, -β2, and -β3, are produced by both glial and neural cells [19]. Previous reports have suggested a relationship between increased TGF-β1 levels and cerebral ischemic injury [20,21]. Following CNS injury, elevated TGF-β levels in astrocytes has been proven to be associated with astrocytic scar formation [22]. Emerging evidence has also demonstrated that TGF-β1 is a crucial mediator in the pathogenesis of several CNS disorders, such as in organization of glial scars in response to injury and in several neurodegenerative disorders [11,15,23].

TGF-βs binds to two serine/threonine kinase receptors which consist of TGF-βRI and TGF-βRII. When a ligand binds, TGF-βRII phosphorylates TGF-βRI and activates Smad-dependent intracellular signaling pathways and thus leads to expression of several genes [24-26]. In addition to activation of Smad-dependent pathways, TGF-β can affect several signal transduction pathways in a Smad-independent manner, such as mitogen-activated protein kinases (MAPKs), including extracellular-signal-related protein kinase (ERK1/2), p38 MAPK, and c-Jun N-terminal kinase (JNK) [12,25,27]. In human gingival and skin fibroblasts, both p38 MAPK and Smad3 cooperate in regulating TGF-β-induced MMP-13 expression, whereas ERK1/2 cooperates with Smad3 in regulating connective tissue growth factor expression [25,28,29]. Recently, increasing evidence has attributed the cellular damage in neurodegenerative disorders to oxidative stress that leads to generation of reactive oxygen species (ROS) that are responsible for brain inflammatory disorders and that have deleterious effects during CNS pathogenic processes [30-32]. TGF-β can stimulate ROS production, which participates in the expression of diverse genes, such as those for MMPs, in the processes of several human diseases like lung fibrosis [33,34]. However, very little information is available concerning the intracellular pathways involved in the effects of TGF-β1 in brain cells.

Recently, several studies have shown that TGF-β1 can up-regulate MMP-9 expression and activity in several cell types such as human skin [35] and corneal epithelial cells [36], implying a crucial role of TGF-β1 in the regulation of MMP-9 in tissue remodeling and wound healing during physiological and pathological processes. The MMP-9 expression is regulated by various mechanisms such as transcriptional and translational regulation in MMP-9 synthesis. The promoter of MMP-9 has been characterized to possess a series of functional enhancer element-binding sites, such as nuclear factor-κB (NF-κB) and activator protein-1 (AP-1), but not in MMP-2 promoter [37,38]. In RBA-1 cells, our previous studies have demonstrated that IL-1β and BK can up-regulate MMP-9 expression via activation of NF-κB [39,40]. However, the possibility of MAPKs and NF-κB activation and their roles in MMP-9 up-regulation and cellular function (migration) induced by TGF-β1 in astrocytes (RBA-1 cells) are poorly defined.

In this study, we investigated the molecular mechanisms and the functional responses underlying TGF-β1-induced MMP-9 expression in RBA-1 cells. These findings indicate that TGF-β1-induced MMP-9 expression via TGF-β receptors is mediated through a ROS-dependent activation of ERK1/2, JNK1/2, and NF-κB pathway, finally leading to cell migration in RBA-1 cells. These results suggest that TGF-β1-induced astrocytic MMP-9 up-regulation might play a key role in physiological and pathological brain tissue remodeling such as wound healing and scar formation.

## Methods

### Materials

DMEM/F-12 medium, fetal bovine serum (FBS), and TRIzol were from Invitrogen (Carlsbad, CA, USA). Hybond C membrane and enhanced chemiluminescence (ECL) western blotting detection system were from GE Healthcare Biosciences (Buckinghamshire, UK). Phospho-(Thr^202^/Tyr^204^)-ERK1/2, phospho-(Ser^176/180^)-JNK1/2, and phospho-(Ser^536^)-p65 antibody kits were from Cell Signaling (Danver, MA, USA). GAPDH antibody was from Biogenesis (Boumemouth, UK). All primary antibodies were diluted at 1:1000 in phosphate-buffered saline (PBS) with 1% BSA (Calbiochem). Actinomycin D, cycloheximide, SB431542, U0126, SB202190, SP600125, helenalin, and Bay11-7082 were from Biomol (Plymouth Meeting, PA, USA). Bicinchoninic acid (BCA) protein assay reagent was from Pierce (Rockford, IL, USA). TGF-β1 was from R&D Systems (Minneapolis, MN, USA). N-acetyl cysteine (NAC), enzymes, XTT assay kit, and other chemicals were from Sigma (St. Louis, MO, USA).

### Rat brain astrocyte culture

RBA-1 cells were used throughout this study. This cell line originated from a primary astrocyte culture of neonatal rat cerebrum and naturally developed through successive cell passages [41]. Staining of RBA-1 with the astrocyte-specific marker, glial fibrillary acid protein (GFAP), showed nearly 95% positive staining. In this study, the RBA-1 cells within 40 passages were used that showed normal cellular morphological characteristics and had steady growth and proliferation in the monolayer system. Cells were cultured and treated as previously described [40]. Primary astrocyte cultures were prepared from the cortex of 6-day-old Sprague-Dawley rat pups as previously described [9]. The purity of primary astrocyte cultures was assessed with the astrocyte-specific marker, GFAP, showing over 95% GFAP-positive astrocytes. The cells were plated on 12-well plates and 10-cm culture dishes for MMP gelatin zymography and RT-PCR, respectively. The culture medium was changed every 3 days.

### MMP gelatin zymography

After TGF-β1 treatment, the culture medium was collected, mixed with equal amounts of non-reduced sample buffer, and electrophoresed on 10% SDS-polyacrylamide gels containing 1 mg/ml gelatin as a protease substrate. Following electrophoresis, gelatinolytic activity was determined as previously described [40]. Mixed human MMP-2 and MMP-9 standards (Chemicon, Temecula, CA, USA) were used as positive controls. Because cleaved MMPs were not reliably detectable, only proform zymogens were quantified. When inhibitors were used, they were added 1 h prior to the application of TGF-β1. Treatment of RBA-1 cells with the pharmacological inhibitors alone had no significant effect on cell viability determined by an XTT assay (data not shown).

### Total RNA extraction and RT-PCR analysis

For RT-PCR analysis of MMP-9 mRNA expression, total RNA was extracted from RBA-1 cells stimulated by TGF-β1 as previously described [40]. The cDNA obtained from 1 μg total RNA was used as a template for PCR amplification. Oligonucleotide primers were designed based on Genbank entries for rat MMP-9 and β-actin. The following primers were used for amplification reaction: for MMP-9: 5'-AGTTTGGTGTCGCGGAGCAC-3' (sense), 5'-TACATGAGCGCTTCCGGCAC-3' (anti-sense); for β-actin: 5'-GAACCCTAAGGCCAACCGTG-3' (sense), 5'-TGGCATAGAGGTCTTTACGG-3' (anti-sense). The PCR amplification was performed in 30 cycles at 55°C, 30 s; 72°C, 1 min; 94°C, 30 s. PCR fragments were analyzed on 2% agarose 1X TAE gel containing ethidium bromide and their size was compared to a molecular weight markers. Amplification of β-actin, a relatively invariant internal reference RNA, was performed in parallel, and cDNA amounts were standardized to equivalent β-actin mRNA levels. These primer sets specifically recognize only the genes of interest as indicated by amplification of a single band of the expected size (754 bp for MMP-9 and 514 bp for β-actin) and direct sequence analysis of the PCR product.

### Cell migration (wound healing) assay

RBA-1 cells were grown to confluence in 6 well plates and starved with serum-free DMEM/F-12 medium for 24 h. The monolayer cells were manually scratched with a pipette tip to create extended and definite scratches in the center of the dishes with a bright and clear field. The detached cells were removed by washing the cells once with PBS. Serum-free DMEM/F-12 medium with or without TGF-β1 was added to each dish as indicated after pretreatment with the inhibitors for 1 h. Images of migratory cells from the scratched boundary were observed and acquired at 48 h with a digital camera and a light microscope (Olympus, Japan). The images shown represent one of three individual experiments.

### Preparation of cell extracts and western blot analysis

Growth-arrested RBA-1 cells were incubated with TGF-β1 at 37°C for the indicated time intervals. The cells were washed with ice-cold PBS, scraped, and collected by centrifugation at 45,000 × g for 1 h at 4°C to yield the whole cell extract, as previously described [40]. Samples were denatured, subjected to SDS-PAGE using a 10% (w/v) running gel, and transferred to nitrocellulose membrane. Membranes were incubated overnight using an anti-phospho-ERK1/2, phospho-JNK1/2, phospho-p65, or GAPDH antibody. Membranes were washed with TTBS four times for 5 min each, incubated with a 1:2000 dilution of anti-rabbit horseradish peroxidase antibody for 1 h. The immunoreactive bands were detected by ECL reagents.

### Measurement of intracellular ROS generation

The peroxide-sensitive fluorescent probe 2',7'-dichlorofluorescein diacetate (DCF-DA) was used to assess the generation of intracellular ROS [42] with minor modifications. RBA-1 cells in monolayers were incubated with RPMI-1640 supplemented with 5 μM DCF-DA for 45 min at 37°C. The supernatant was removed and replaced with fresh RPMI-1640 media before stimulation with TGF-β1. Relative fluorescence intensity was recorded over time (3 to 60 minutes) by using a fluorescent plate reader (Thermo, Appliskan) at an excitation wavelength of 485 nm and emission was measured at a wavelength of 530 nm.

### Plasmid construction, transient transfection, and promoter activity assays

The dominant negative plasmids encoding ERK1 (ΔERK1), ERK2 (ΔERK2), p38 (Δp38), and JNK (ΔJNK) were kindly provided by Dr. K.L. Guan (Department of Biological Chemistry, University of Michigan, MI), Dr. J. Han (The Scripps Research Institute, La Jolla, CA, USA), and C.C. Chen (Department of Pharmacology, National Taiwan University, Taipei, Taiwan), respectively. The rat MMP-9 promoter was constructed as previously described [43] with some modifications. The upstream region (-1280 to +108) of the rat MMP-9 promoter was cloned into the pGL3-basic vector containing the luciferase reporter system. Introduction of a double-point mutation into the NF-κB-binding site (κB domain; GGAATTCC to GGAATTGG) to generate pGL-MMP-9-DκB (mt-κB-MMP-9) was performed using the following (forward) primer: 5'-GGGTTGCCCCGTGGAATTGGCCCAAATCCTGC-3' (corresponding to a region from -572 to -541). The underlined nucleotides indicate the positions of substituted bases. All plasmids were prepared by using QIAGEN plasmid DNA preparation kits. The MMP-9 promoter reporter constructs were transfected into RBA-1 cells using the Lipofetamine™RNAiMAX reagent according to the instructions of manufacture (Invitrogen, Carlsbad, CA). The transfection efficiency (~60%) was determined by transfection with enhanced EGFP. To assess promoter activity, cells were collected and disrupted by sonication in lysis buffer (25 mM Tris-phosphate, pH 7.8, 2 mM EDTA, 1% Triton X-100, and 10% glycerol). After centrifugation, aliquots of the supernatants were tested for luciferase activity using a luciferase assay system. Firefly luciferase activities were standardized to b-galactosidase activity.

### Analysis of data

All data were estimated using GraphPad Prism Program (GraphPad, San Diego, CA, USA). Quantitative data were analyzed by one-way ANOVA followed by Tukey's honestly significant difference tests between individual groups. Data were expressed as mean ± SEM. A value of *P *< 0.05 was considered significant.

## Results

### TGF-β1 induces *de novo *synthesis of MMP-9 and cell migration in RBA-1 cells

**Figure 1:**
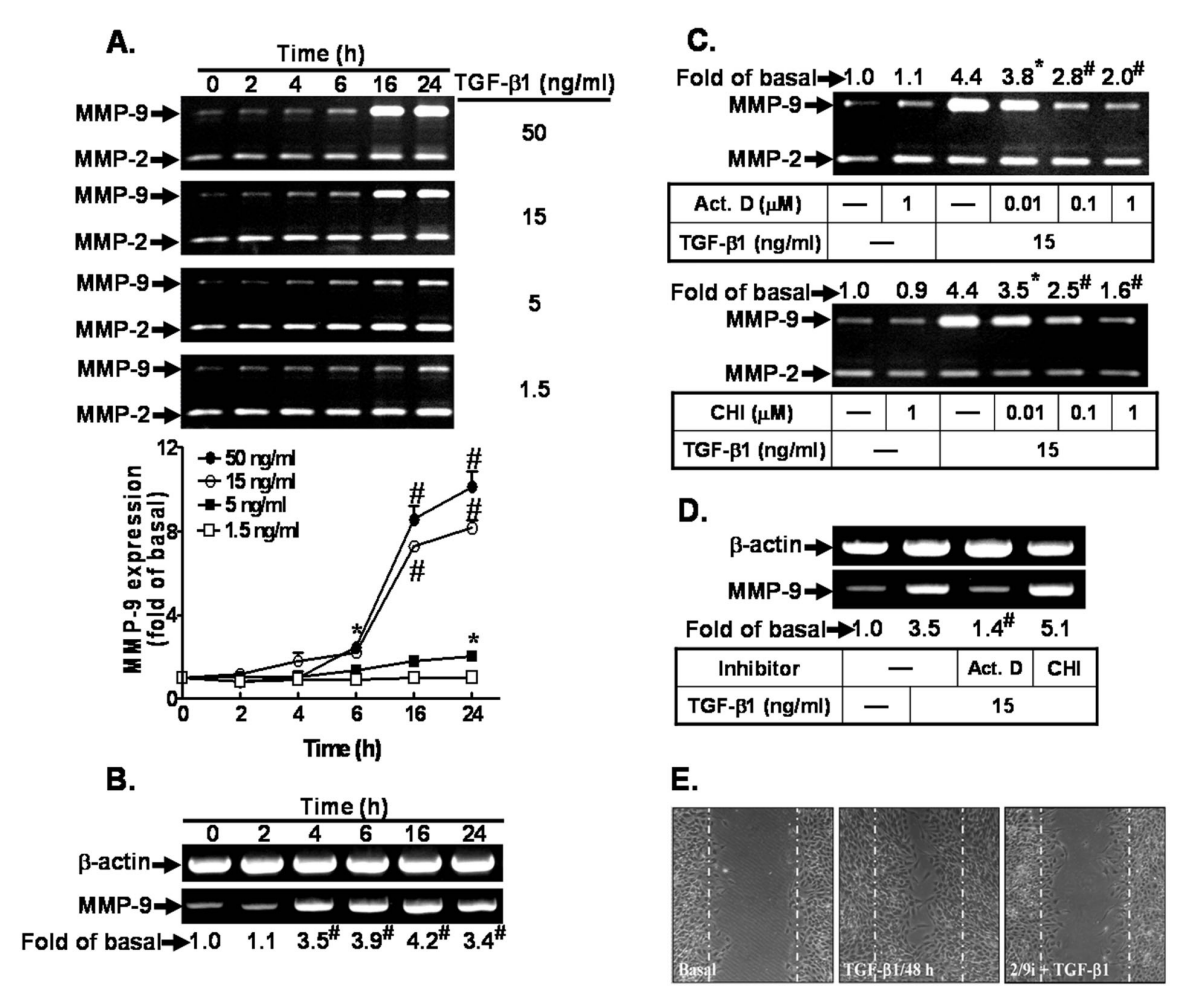
**TGF-β1 induces *de novo *synthesis of MMP-9 and cell migration in RBA-1 cells**. (A) Time and concentration dependence of TGF-β1-induced MMP-9 expression. Cells were incubated with various concentrations of TGF-β1 for the indicated time intervals. The conditioned media were collected and analyzed by gelatin zymography. The levels of TGF-β1-induced MMP-9 expression were quantified (right panel). (B) Cells were treated with TGF-β1 (15 ng/ml) for the indicated time intervals. Total RNA was collected and analyzed by RT-PCR. (C) Cells were pretreated with actinomycin D (Act. D, upper panel) or cycloheximide (CHI, lower panel) for 1 h and then incubated with TGF-β1 for 16 h. (D) Cells were pretreated with or without Act.D (1 μM) or CHI (1 μM) before exposure to TGF-β1 for 6 h. The conditioned media and total RNA were collected and analyzed by gelatin zymography (C) and RT-PCR (D). (E) For cell migration, cells were plated on 6-well plates and grew to confluence. Cells were manually scratched with a pipette tip, and then incubated with or without TGF-β1 (15 ng/ml) for 48 h after pretreatment with MMP-2/9 inhibitor (2/9i, 3 μM) for 1 h. Phase contrast images of RBA-1 cells were taken at 48 h in response to TGF-β1. Data are expressed as mean ± SEM (A) or mean (B, C, D) of three independent experiments (n = 3). *P < 0.05; ^#^*P *< 0.01, as compared with the cells exposed to vehicle (A, B) or TGF-β1 (C, D) alone. The image represents one of three similar experiments (n = 3).

To investigate the effects of TGF-β1 on MMP-9 expression, RBA-1 cells were treated with various concentrations of TGF-β1 for the indicated time intervals. The condition media were collected and analyzed by gelatin zymography. As shown in Figure 1A, TGF-β1 induced MMP-9 expression in a time- and concentration-dependent manner. There was an apparent up-regulation within 16 h and sustained over 24 h. In contrast, the expression of MMP-2 was not significantly changed during incubation with TGF-β1 (Figure 1A). To further examine whether the increase of MMP-9 expression by TGF-β1 resulted from the induction of MMP-9 mRNA expression, a RT-PCR analysis was performed. The data show that TGF-β1 time-dependently induced MMP-9 mRNA expression in RBA-1 cells (Figure 1B), whereas the expression of a housekeeping gene β-actin (as an internal control) mRNA was not changed. There was a significant increase in MMP-9 mRNA within 4 h and sustained over 24 h during the period of observation. Moreover, to determine whether the TGF-β1-induced MMP-9 expression is dependent on *de novo *protein synthesis, the cells were exposed to TGF-β1 in the absence or presence of actinomycin D (Act. D) or cycloheximide (CHI) at a dose known to inhibit transcription or protein synthesis [44], respectively. The results show that TGF-β1-induced MMP-9 expression was significantly attenuated by pretreatment with either Act. D (Figure 1C, upper panel) or CHI (Figure 1C, lower panel) in a concentration-dependent manner. Moreover, TGF-β1-induced MMP-9 mRNA accumulation was attenuated by pretreatment with Act.D but not with CHI (Figure 1D). Moreover, to demonstrate the functional activity of MMP-9 expression induced by TGF-β1, we evaluated *in vitro *cell migration of RBA-1 by a cell migration assay. After 48 h of TGF-β1 incubation, the images show that TGF-β1-enhanced cell migration was blocked by pretreatment with the inhibitor of MMP-2/9 activity (2/9i) (Figure 1E), suggesting that up-regulation of MMP-9 and its activity are required for enhancing RBA-1 cell migration induced by TGF-β1.

### TGF-β1 induces MMP-9 expression and cell migration via a TGF-β type I receptor

**Figure 2:**
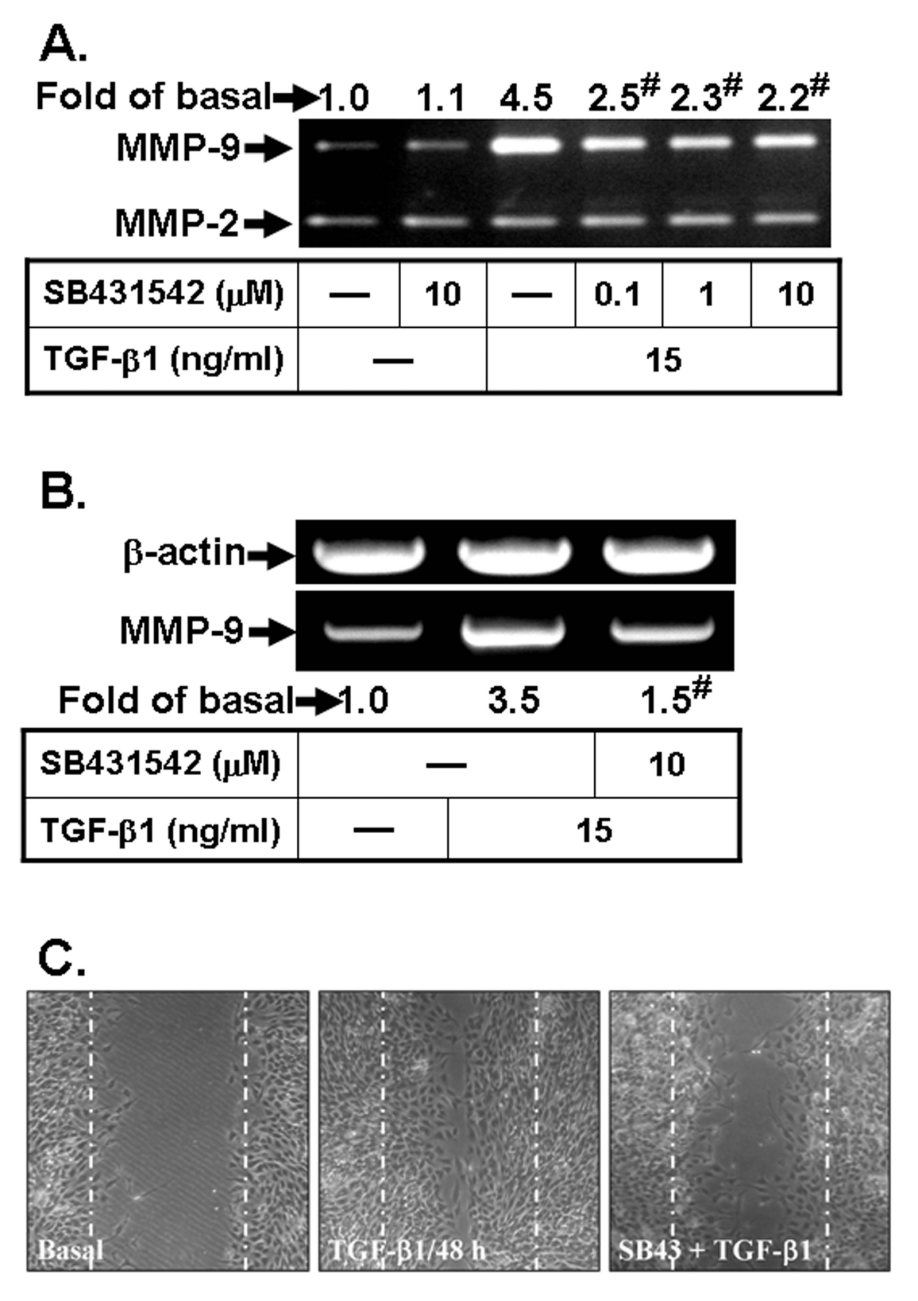
**TGF-β1 induces MMP-9 expression and cell migration via a TGF-β1 receptor signaling**. (A) Cells were pretreated with or without SB431542 for 1 h before exposure to TGF-β1 for 16 h. The conditioned media were collected and analyzed by gelatin zymography. (B) Cells were pretreated with SB431542 (10 μM) before exposure to TGF-β1 for 6 h. Total RNA was collected and analyzed by RT-PCR. (C) For cell migration, cells were pretreated with SB431542 (10 μM) for 1 h and then incubated with TGF-β1 (15 ng/ml) for 48 h. Representative phase contrast images are shown for 48 h (n = 3). Data are expressed as mean of at least three independent experiments (n = 3). ^#^*P *< 0.01, as compared with the cells exposed to TGF-β1 alone. The figure represents one of three individual experiments.

SB431542, a selective inhibitor of TGF-β Type I receptor (TGF-βRI), has been shown to abrogate TGF-β1-mediated expression of several genes in different cell types [45-47]. Thus, we examined whether TGF-β1 induced MMP-9 expression via TGF-βRI, a selective TGF-βRI antagonist SB431542 was used for this purpose. The data reveal that blockade of TGF-βRI by SB431542 attenuated both TGF-β1-induced MMP-9 protein (Figure 2A) and mRNA (Figure 2B) expression. Moreover, the involvement of TGF-βRI in TGF-β1-induced cell migration was characterized by a cell migration assay. The image data show that pretreatment with SB431542 significantly attenuated TGF-β1-enhanced cell migration (Figure 2C). These results demonstrate that TGF-βRI-mediated MMP-9 induction is essential for enhancing RBA-1 cell migration.

### TGF-β1-induced MMP-9 expression is mediated through ERK1/2

**Figure 3:**
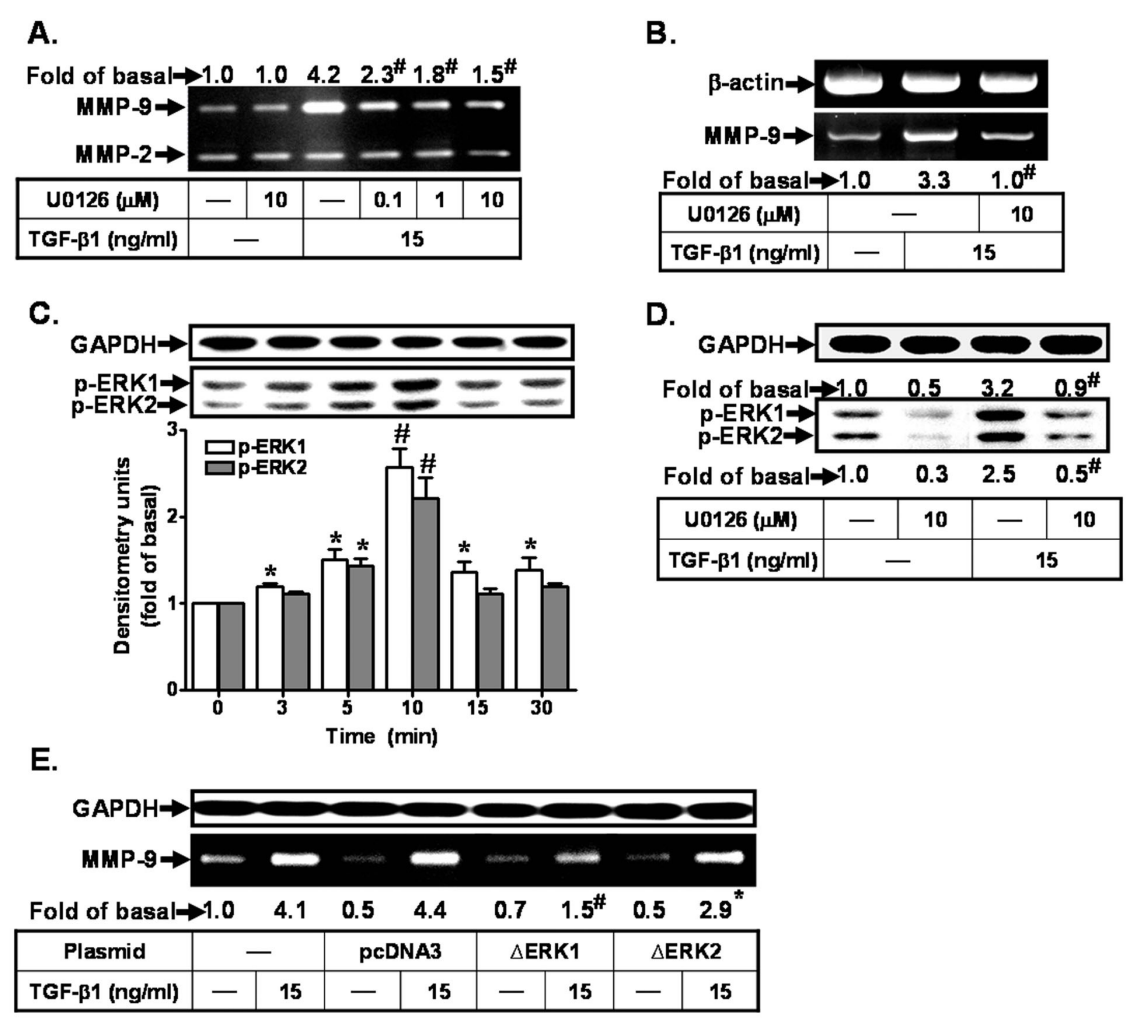
**Involvement of ERK1/2 in TGF-β1-induced MMP-9 expression in RBA-1 cells**. (A) Cells were treated with TGF-β1 (15 ng/ml) for 16 h in the absence or presence of U0126. (B) Cells were pretreated with U0126 (10 mM) before exposure to TGF-β1 for 6 h. Total RNA was collected and analyzed by RT-PCR. (C) Time dependence of TGF-β1-stimulated ERK1/2 phosphorylation, cells were incubated with TGF-β1 (15 ng/ml) for the indicated time intevals. (D) Cells were treated with TGF-β1 (15 ng/ml) for 10 min in the absence or presence of U0126 (10 μM). The whole cell lysates (C, D) were subjected to 10% SDS-PAGE and analyzed using an anti-phospho-ERK1/2 or anti-GAPDH (as an internal control) antibody. (E) Cells were transfected with an empty vector (pcDNA3, as a control), a dominant negative mutant of ERK1 (ΔERK1) or ERK2 (ΔERK2) for 24 h, and then exposed to TGF-β1 for 16 h. The conditioned media (A, E) were analyzed gelatin zymorgraphy. Data are expressed as mean ± SEM (C) or mean (A, B, D, E) of at least three independent experiments (n = 3). *P < 0.05; ^#^*P *< 0.01, as compared with the cells exposed to vehicle (C) or TGF-β1 (A, B, D, E) alone. The figure represents one of three individual experiments.

Accumulating evidence suggests that activation of MAPK family, including ERK1/2, JNK1/2, and p38 MAPK, by TGF-β1 modulates cellular functions of different cell types in CNS [48,49]. First, to investigate the role of ERK1/2 in TGF-β1-induced MMP-9 expression in RBA-1, cells were pretreated with an inhibitor of MEK1/2, an upstream kinase of ERK1/2, U0126 for 1 h and then incubated with TGF-β1 for 16 h. As shown in Figure 3A, pretreatment with U0126 significantly inhibited TGF-β1-induced MMP-9 expression in a concentration-dependent manner. Moreover, pretreatment with U0126 (10 μM) also blocked TGF-β1-induced MMP-9 mRNA accumulation (Figure 3B). To determine whether ERK1/2 phosphorylation was necessary for the induction of MMP-9 expression in response to TGF-β1, activation of ERK1/2 was assayed using an antibody specific for the phosphorylated form of ERK1/2. The data show that TGF-β1 stimulated the phosphorylation of ERK1/2 in a time-dependent manner with a maximal response obtained within 10 min (Figure 3C). In addition, pretreatment with U0126 (10 μM) completely inhibited TGF-β1-stimulated ERK1/2 phosphorylation (Figure 3D). To further ensure the role of ERK1/2 in TGF-β1-induced MMP-9 expression, cells were transfected with dominant negative mutant of either ERK1 (ΔERK1) or ERK2 (ΔERK2) and then incubated with TGF-β1 for 16 h. The data show that transfection with either ΔERK1 or ΔERK2 significantly attenuated TGF-β1-induced MMP-9 expression (Figure 3E), indicating that ERK1/2 is involved in TGF-β1-induced MMP-9 expression in RBA-1 cells.

### JNK1/2, but not p38 MAPK, is involved in TGF-β1-induced MMP-9 expression

**Figure 4:**
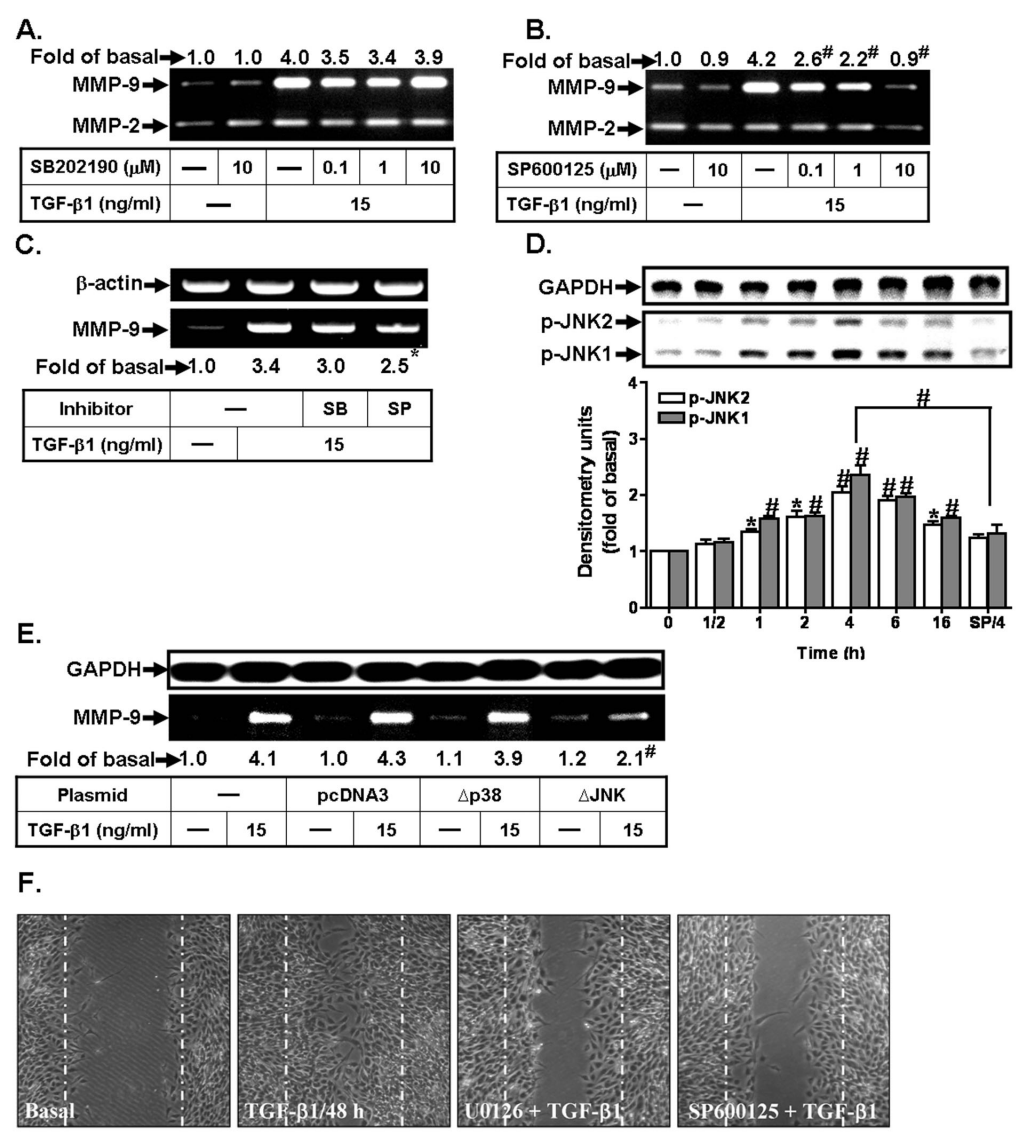
**TGF-β1-induced MMP-9 expression is mediated through JNK1/2, but not p38 MAPK**. Cells were treated with TGF-β1 (15 ng/ml) for 16 h in the absence or presence of (A) SB202190 or (B) SP600125. (C) Cells were pretreated with SB202190 (SB, 10 μM) or SP600125 (SP, 10 μM) before exposure to TGF-β1 for 6 h. Total RNA was collected and analyzed by RT-PCR. (D) Time dependence of TGF-β1-stimulated JNK1/2 phosphorylation, cells were incubated with TGF-β1 (15 ng/ml) for the indicated times. Moreover, cells were treated with TGF-β1 for 4 h in the presence of SP600125 (SP/4, 10 μM). (E) Cells were transfected with an empty vector (pcDNA3, as a control) or dominant negative mutant of p38 MAPK (Δp38) or JNK (ΔJNK) for 24 h, and then exposed to TGF-β1 for 16 h. (F) For cell migration, cells were pretreated with U0126 (10 μM) or SP600125 (10 μM) for 1 h and then incubated with TGF-β1 (15 ng/ml) for 48 h. The image is representative of three similar experiments (n = 3). The conditioned media (A, B, E) were analyzed gelatin zymorgraphy, and the whole cell lysates (D) were analyzed using an anti-phospho-JNK1/2 or anti-GAPDH (as an internal control) antibody. Data are expressed as mean ± SEM (D) or mean (A, B, C, E) of at least three independent experiments (n = 3). *P < 0.05; ^#^*P *< 0.01, as compared with the cells exposed to vehicle (D) or TGF-β1 (A, B, D, E) alone. The figure represents one of three individual experiments.

Next, we investigated the roles of p38 MAPK and JNK1/2 in TGF-β1-induced MMP-9 expression in RBA-1, cells were pretreated with the inhibitor of either p38 MAPK (SB202190) or JNK1/2 (SP600125) for 1 h and then incubated with TGF-β1 for 16 h. The data show that pretreatment with SB202190 had no significant effect on TGF-β1-induced MMP-9 expression (Figure 4A). Pretreatment with SP600125 significantly attenuated TGF-β1-induced MMP-9 expression (Figure 4B). TGF-β1-induced MMP-9 mRNA expression was also inhibited by pretreatment with SP600125, but not SB202190 (Figure 4C), suggesting that TGF-β1-induced MMP-9 gene expression is mediated through JNK1/2, but not p38 MAPK. To determine whether JNK1/2 phosphorylation was necessary for the induction of MMP-9 expression in response to TGF-β1, the activation of JNK1/2 was assayed using an antibody specific for the phosphorylated form of JNK1/2. The data reveal that TGF-β1 stimulated the phosphorylation of JNK1/2 in a time-dependent manner with a maximal response obtained within 4 h (Figure 4D). Pretreatment with SP600125 (10 μM) significantly blocked TGF-β1-stimulated JNK1/2 phosphorylation (Figure 4D). Similarly, TGF-β1 stimulated p38 MAPK phosphorylation, which was attenuated by pretreatment with SB202190 (10 μM) (data not shown). To further ensure the role of JNK in TGF-β1-induced MMP-9 expression, cells were transfected with dominant negative mutant of either p38 MAPK (Δp38) or JNK (ΔJNK) and then incubated with TGF-β1 for 16 h. The data show that transfection with ΔJNK markedly inhibited TGF-β1-induced MMP-9 expression, whereas transfection with Δp38 had no apparent change in TGF-β1-induced MMP-9 expression (Figure 4E). These results demonstrate that JNK1/2 is also involved in TGF-β1-induced MMP-9 expression in RBA-1 cells. For cell migration, pretreatment with either U0126 or SP600125 significantly attenuated TGF-β1-induced astrocytic migration (Figure 4F), indicating that TGF-β1 induces cell migration via ERK1/2 and JNK pathways in RBA-1 cells.

### Involvement of ROS-dependent ERK1/2 and JNK1/2 pathways in TGF-β1-induced MMP-9 expression

**Figure 5:**
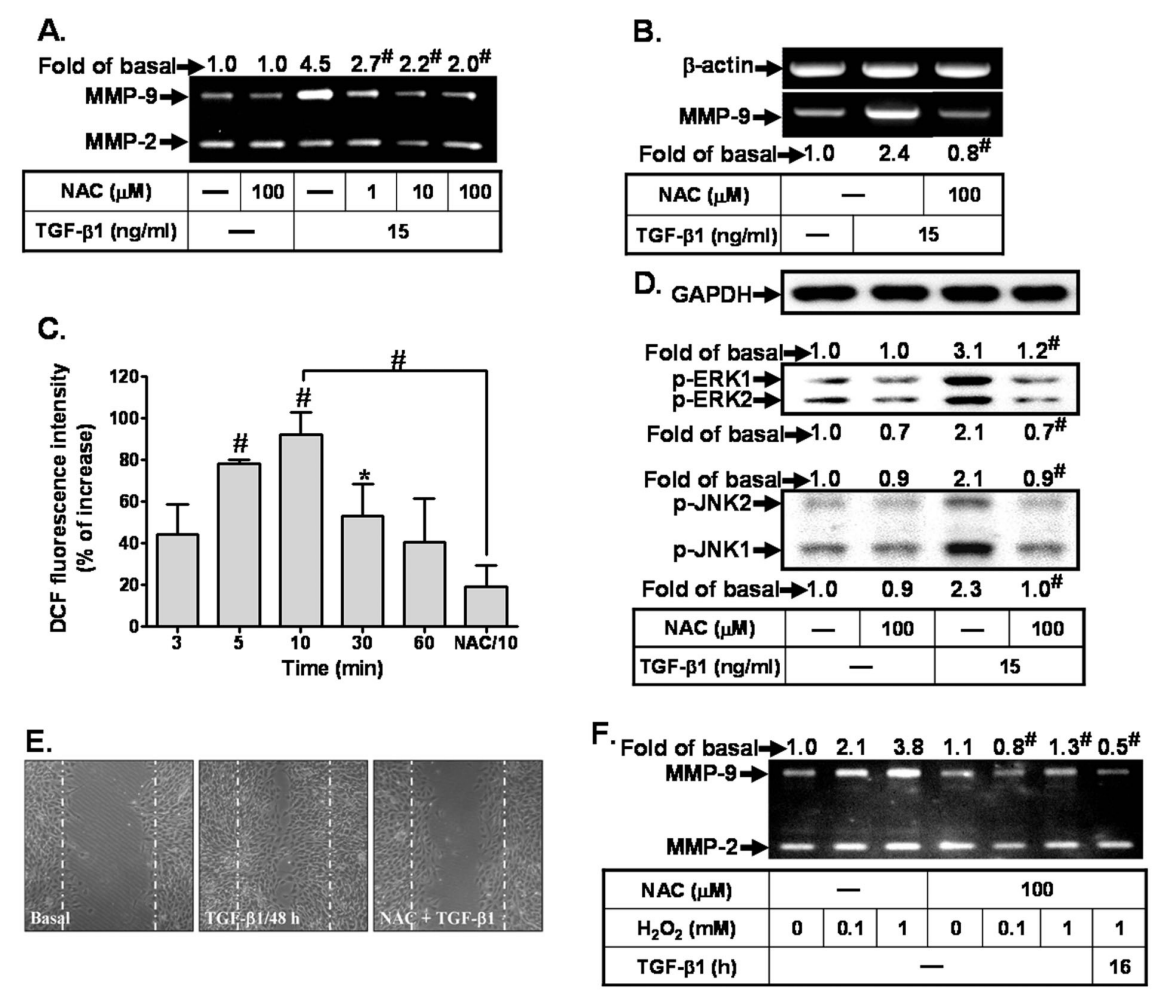
**ROS-dependent MAPK signaling is essential for TGF-β1-induced MMP-9 expression and cell migration in RBA-1 cells**. (A) Cells were treated with TGF-β1 (15 ng/ml) for 16 h in the absence or presence of N-acetyl cysteine (NAC). (B) Cells were pretreated with NAC (100 μM) before exposure to TGF-β1 for 6 h. The conditioned media and total RNA were collected and analyzed by gelatin zymography (A) and RT-PCR (B). (C) Time dependence of TGF-β1-stimulated intracellular ROS generation, RBA-1 cells were incubated with the peroxide-sensitive fluorescent probe DCF-DA (5 μM) for 45 min, followed by stimulation with TGF-β1 (15 ng/ml) for the indicated time intervals. Moreover, cells were treated with TGF-β1 for 10 min in the presence of NAC (NAC/10, 100 μM). The DCF fluorescence intensity of cells was determined. (D) Cells were pretreated with or without NAC (100 μM) for 1 h before exposure to TGF-β1 for 10 min (for ERK1/2) or 4 h (for JNK1/2). Whole cell lysates were analyzed using an anti-phospho-ERK1/2, anti-phospho-JNK1/2, or anti-GAPDH (as an internal control) antibody. (E) For cell migration, cells were pretreated with NAC (100 μM) for 1 h and then incubated with TGF-β1 (15 ng/ml) for 48 h. Representative phase contrast images are shown for 48 h (n = 3). (F) Cells were pretreated with or without NAC (100 μM) for 1 h before exposure to H_2_O_2 _(0.1 or 1 μM) for 16 h or H_2_O_2 _(100 μM) and TGF-β1 for 16 h. Data are expressed as mean ± SEM (C) or mean (A, B, D, F) of three independent experiments (n = 3). *P < 0.05; ^#^*P *< 0.01, as compared with the cells exposed to vehicle (C) or TGF-β1 (A, B, D, F) alone. The figure represents one of three similar experiments.

Recently, several reports have demonstrated that increasing ROS production contributes to expression of several genes such as MMP-9 in different cell types [50-52]. To examine whether ROS participated in TGF-β1-induced MMP-9 expression, cells were pretreated with N-acetyl cysteine (NAC, an ROS scavenger) for 1 h and then incubated with TGF-β1 for 16 h. Our results show that pretreatment with NAC reduced TGF-β1-induced MMP-9 expression (Figure 5A) and its mRNA accumulation (Figure 5B), implying that ROS may contribute to induction of MMP-9 by TGF-β1 in RBA-1 cells. To determine whether generation of ROS was involved in TGF-β1-induced MMP-9 expression in RBA-1 cells, a fluorescent probe DCF-DA was used to determine the generation of ROS in these cells. RBA-1 cells were labeled with DCF-DA, incubated with TGF-β1 for the indicated time intervals, and the fluorescence intensity (relative DCF fluorescence) was measured at 485-nm excitation and 530-nm emission. The data reveal that TGF-β1 stimulated intracellular ROS generation in a time-dependent manner with a maximal response within 10 min and sustained over 60 min (Figure 5C). Furthermore, TGF-β1-stimulated ROS generation was markedly attenuated by pretreatment with NAC (Figure 5C), demonstrating that NAC is an efficient ROS scavenger. Next, to determine whether TGF-β1-induced MAPK phosphorylation occurs via a ROS-dependent pathway, we pretreated cells with NAC for 1 h and then incubated them with TGF-β1 for 10 min (for ERK1/2 phosphorylation) or 4 h (for JNK1/2 phosphorylation). These results show that pretreatment with NAC (100 μM) significantly reduced TGF-β1-stimulated phosphorylation of ERK1/2 and JNK1/2 in RBA-1 cells (Figure 5D). In addition, the role of ROS in TGF-β1-induced cell migration was assessed by a cell migration assay. The imaging data show that TGF-β1-induced cell migration is attenuated by pretreatment with NAC (Figure 5E). Furthermore, to demonstrate the direct role of ROS in MMP-9 up-regulation, cells were directly exposed to various concentrations (0.1 and 1 mM) of H_2_O_2 _or to combination of 1 mM of H_2_O_2 _and 15 ng/ml of TGF-β1 for 24 h. The data show that exposure of cells to H_2_O_2 _concentration-dependently induced MMP-9 expression which was blocked by pretreatment with NAC (Figure 5F), suggesting that ROS play a critical role in up-regulation of MMP-9 in RBA-1 cells. These results suggest that ROS-dependent ERK1/2 and JNK1/2 cascades may contribute to TGF-β1-induced MMP-9 expression and cell migration in RBA-1 cells.

### NF-κB is required for TGF-β1-induced MMP-9 expression and cell migration in RBA-1 cells

**Figure 6:**
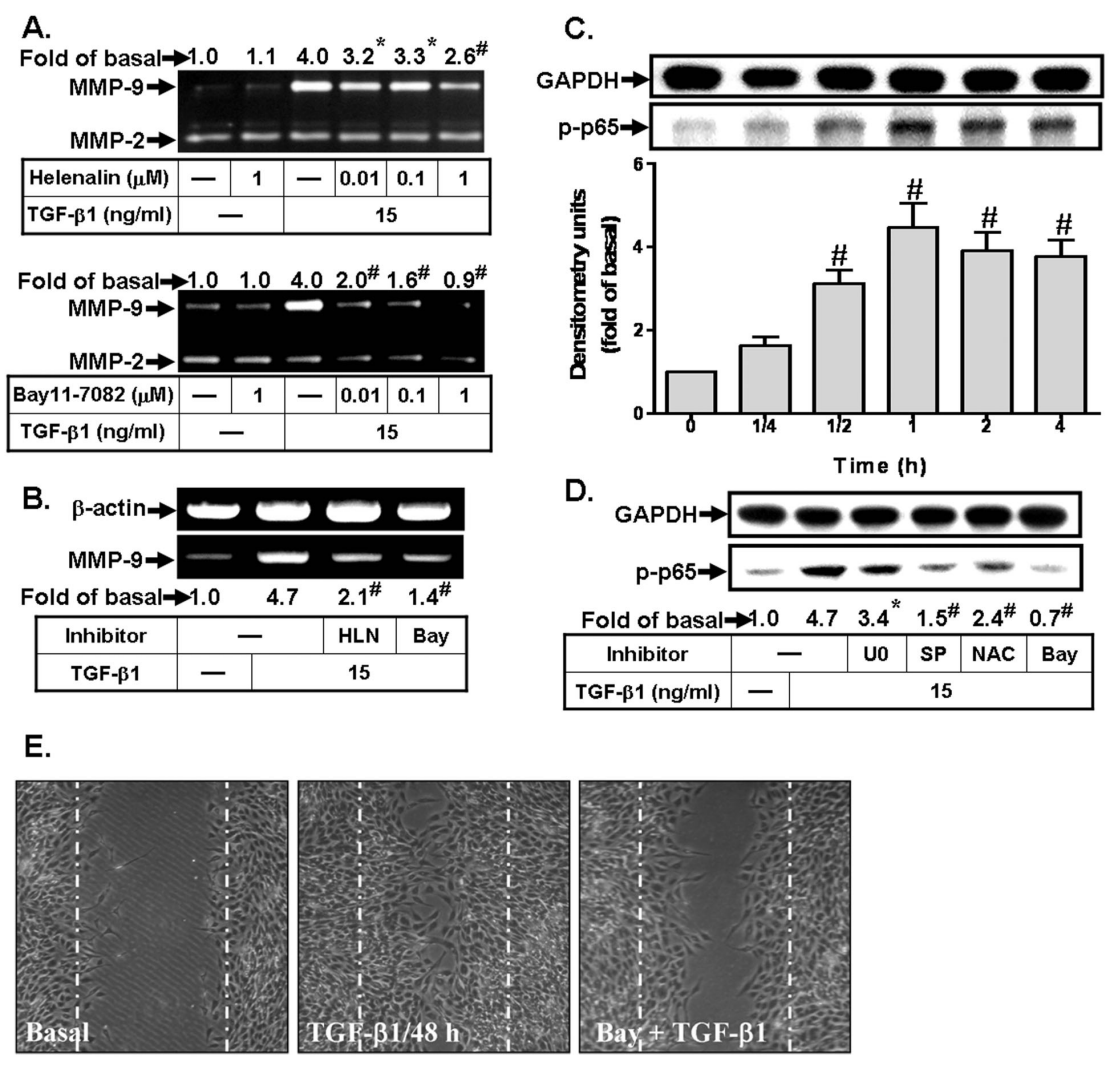
**NF-κB is involved in TGF-β1-induced MMP-9 expression and cell migration in RBA-1 cells**. (A) Cells were treated with TGF-β1 (15 ng/ml) for 16 h in the absence or presence of helenalin or Bay11-7082. (B) Cells were pretreated with helenalin (HLN, 1 μM) or Bay11-7082 (Bay, 1 μM) before exposure to TGF-β1 for 6 h. The conditioned media and total RNA were collected and analyzed by gelatin zymography (A) and RT-PCR (B). (C) Time dependence of TGF-β1-stimulated NF-κB p65 phosphorylation, cells were incubated with TGF-β1 (15 ng/ml) for the indicated time intervals. (D) Cells were pretreated with U0126 (U0, 10 μM), SP600125 (SP, 10 μM), NAC (100 μM), or Bay11-7082 (Bay, 1 μM) for 1 h before exposure to TGF-β1 for 1 h. Whole cell lysates were analyzed by western blotting using an anti-phospho-NF-κB-p65 antibody. (E) For cell migration, cells were pretreated with Bay11-7082 (1 μM) for 1 h and then incubated with TGF-β1 (15 ng/ml) for 48 h. Representative phase contrast images are shown for 48 h (n = 3). Data are expressed as mean ± SEM (C) or mean (A, B, D) of three independent experiments (n = 3). *P < 0.05; ^#^*P *< 0.01, as compared with the cells exposed to vehicle (C) or TGF-β1 (A, B, D) alone. The figure represents one of three similar experiments.

Recent findings have suggested that NF-κB is a fundamental transcription factor for induction of several genes such as MMP-9 in astrocytes [9,53]. Moreover, as shown in Figures 1C and 1D, we found that TGF-β1 induces MMP-9 expression at the transcriptional level. The MMP-9 gene promoter with potential binding elements is required for recognition of transcription factors including NF-κB [37]. On the other hand, the NF-κB family is considered to be an essential regulator of both cellular and inflammatory activities [54]. In astrocytes, TGF-β1 has been shown to stimulate NF-κB activation, associated with astrocyte activation during CNS injury [55]. Thus, we examined whether NF-κB was required for induction of MMP-9 by TGF-β1 in RBA-1 cells. First, cells were pretreated with the selective NF-κB inhibitors, helenalin and Bay11-7082, which block activation of NF-κB signaling [56], and then incubated with TGF-β1 for 16 h. The zymographic data show that pretreatment with either helenalin or Bay11-7082 significantly attenuated TGF-β1-induced MMP-9 expression (Figure 6A) and mRNA accumulation (Figure 6B), suggesting the involvement of NF-κB in TGF-β1-induced MMP-9 expression in RBA-1 cells. To further ensure that activation of NF-κB is involved in signaling stimulated by TGF-β1, the phosphorylation of NF-κB p65 was determined by western blot using an anti-phospho-p65 NF-κB antibody. As shown in Figure 6C, TGF-β1 stimulated phosphorylation of NF-κB p65 in a time-dependent manner, which was inhibited by pretreatment with U0126 (10 μM), SP600125 (10 μM), NAC (100 μM), or Bay11-7082 (1 μM) (Figure 6D), indicating that TGF-β1-stimulated NF-κB signaling is mediated through ROS-dependent ERK1/2 and JNK1/2 cascades in RBA-1 cells. Furthermore, the cell migratory images show that pretreatment with Bay11-7082 inhibited TGF-β1-induced RBA-1 cell migration (Figure 6E). These results demonstrate that NF-κB is necessary for TGF-β1-induced MMP-9 expression and cell migration in RBA-1 cells.

### Involvement of NF-κB binding site in regulation of the rat MMP-9 promoter by TGF-β1

**Figure 7:**
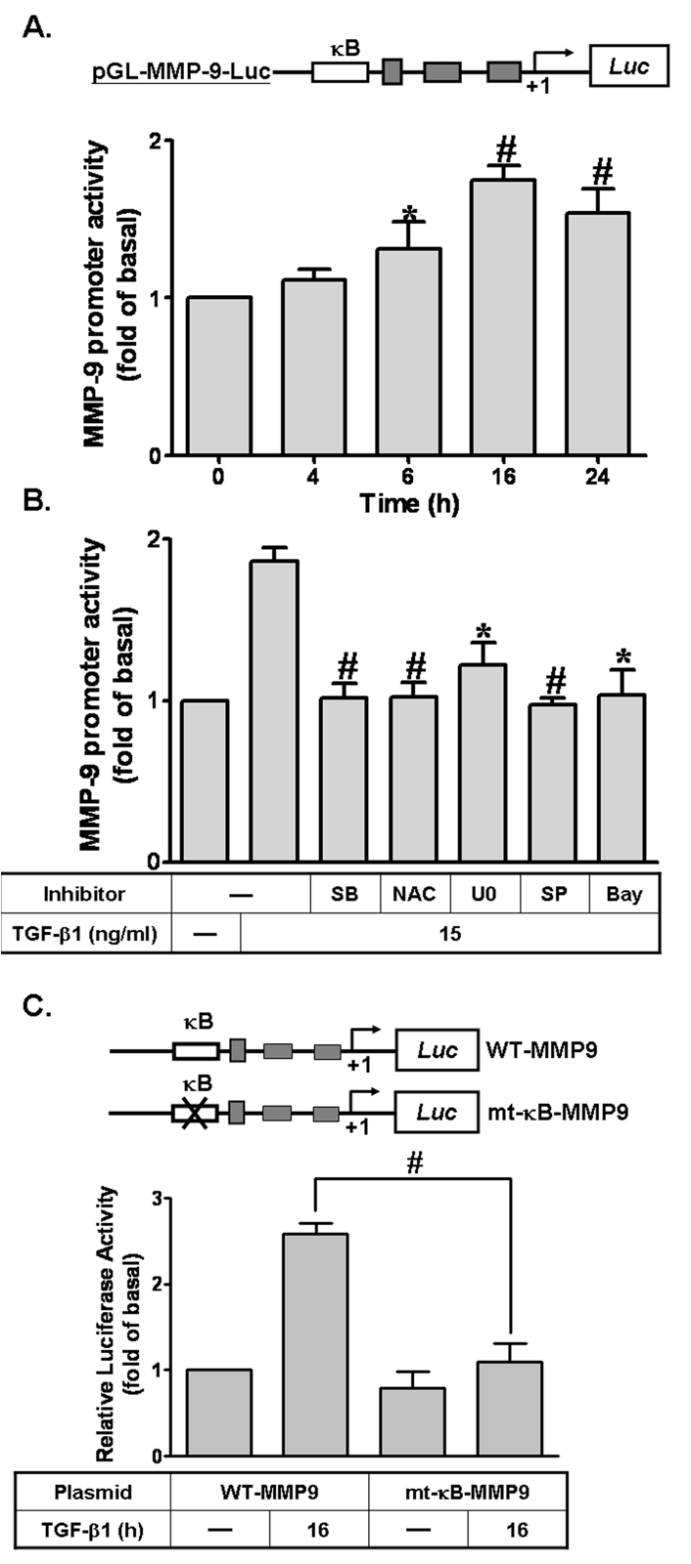
**The ROS/MAPKs-dependent NF-κB cascade is required for TGF-β1-induced MMP-9 promoter activity**. (A) Schematic representation of a 5'-promoter regions of the rat MMP-9 gene fused to the pGL-luciferase reporter gene (pGL-MMP-9-Luc). The translational start site (+1) of the *luciferase *reporter gene is indicated by an arrow. RBA-1 cells were transiently cotransfected with pGL-MMP9-Luc and pGal encoding for b-galactosidase. After transfection, cells were treated with TGF-β1 (15 ng/ml) for the indicated time intervals. (B) Cells were pretreated with SB431542 (SB43, 10 μM), NAC (100 μM), U0126 (10 μM), SP600125 (SP, 10 μM), or Bay11-7082 (Bay, 1 μM) for 1 h, and then incubated with TGF-β1 for 16 h. (C) Activation of wild-type (WT) and NF-κB-point-mutated (mt-κB) MMP-9 promoter constructs by TGF-β1. Schematic representation of the different MMP-9-luciferase constructs, either wild-type (WT) or modified by single-point mutation of the NF-κB binding site (upper panel). After overnight cotransfection and incubation with TGF-β1 for 16 h, promoter activities of different MMP-9-promoter constructs were measured as relative MMP-9 promoter activity to b-galactosidase. The relative increase in MMP-9 promoter activity induced by TGF-β1 normalized to that of un-stimulated cells is indicated as fold increase. Data are expressed as mean ± SEM of at least three independent experiments (n = 3). *P < 0.05; ^#^*P *< 0.01, as compared with the cells exposed to vehicle (A) or TGF-β1 (B, C) alone.

We have found that TGF-β1 stimulates activation of NF-κB. Next, we examined whether the binding of NF-κB to its promoter binding element is essential for TGF-β1-induced MMP-9 gene regulation. The rat MMP-9 promoter luciferase reporter was constructed and its activity was evaluated by a promoter-luciferase activity assay. The rat MMP-9 promoter was constructed into a pGL3-basic vector containing a luciferase reporter system (as illustrated in Figure 7A, upper part; pGL-MMP-9-Luc), which possesses several putative recognition elements for a variety of transcription factors including NF-κB family. Thus, to determine the effect of TGF-β1 on the MMP-9 promoter activity, cells were transfected with a pGL-MMP-9-Luc construct and then incubated with TGF-β1 for the indicated time intervals. As shown in Figure 7A, TGF-β1 increased the MMP-9 promoter activity in a time-dependent manner. A maximal response was obtained within 16 h, which was significantly inhibited by pretreatment with the inhibitor of TGF-βRI (SB431542), MEK1/2 (U0126), JNK1/2 (SP600125), NF-κB (Bay11-7082), or an anti-oxidant (NAC) (Figure 7B). To further ensure that NF-κB mediated TGF-β1-induced MMP-9 promoter activity through binding to their regulatory elements within the MMP-9 promoter region, wild-type (WT) MMP-9 promoter, mutated by a single-point mutation of the κB binding site (mt-κB-MMP-9), was constructed (as indicated in Figure 7C, upper part). As shown in Figure 7C, TGF-β1-stimulated MMP-9 promoter activity was significantly attenuated in RBA-1 cells transfected with mt-κB-MMP-9, indicating that the κB element is essential for TGF-β1-induced MMP-9 promoter activity. These results further confirm that TGF-β1 induces MMP-9 promoter activity via enhanced NF-κB binding to the κB element of the MMP-9 promoter in RBA-1 cells.

**Figure 8:**
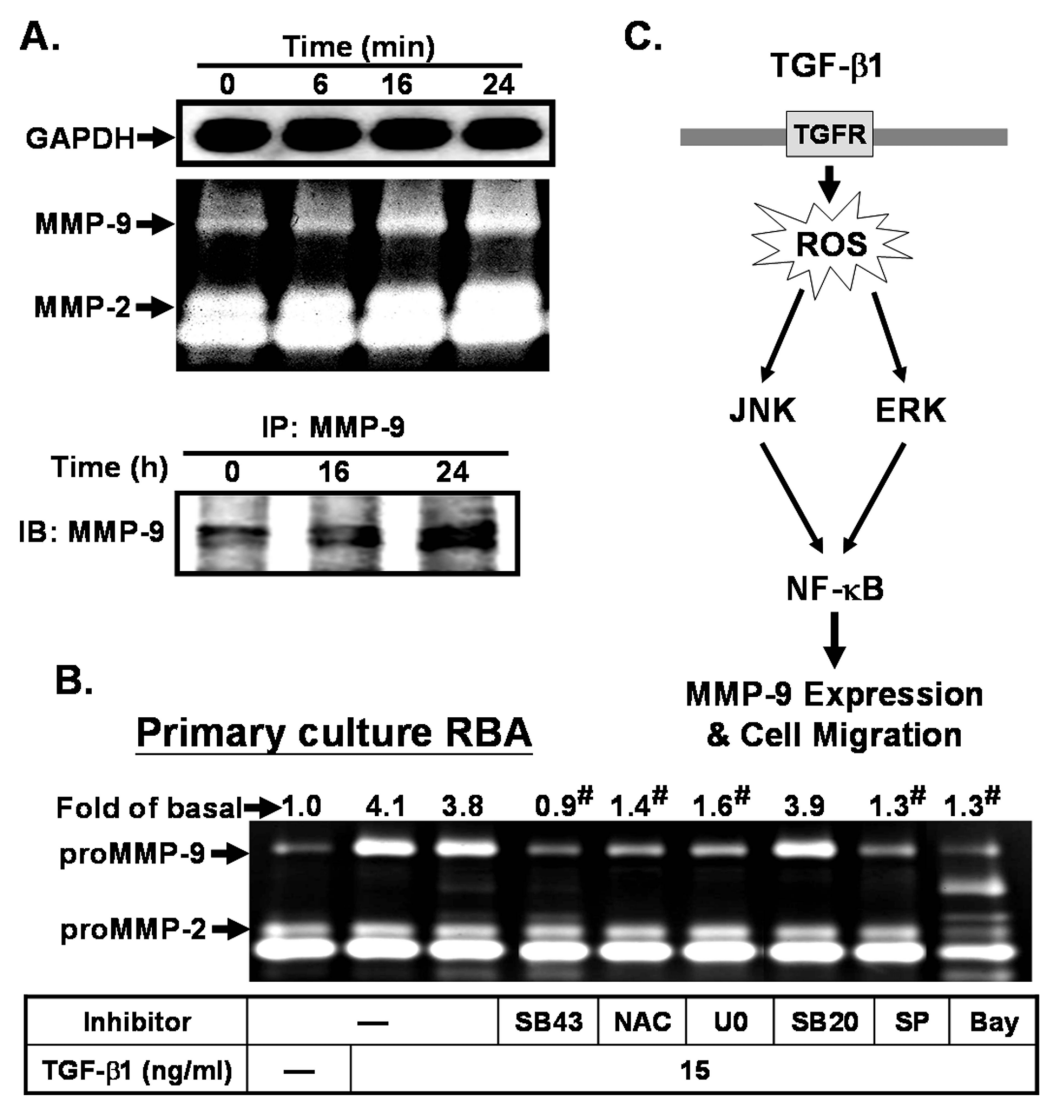
**TGF-β1 induces MMP-9 expression and activation in the rat primary cultured astrocytes**. (A) Time dependence of TGF-β1-induced MMP-9 expression and activation. Cells were treated with TGF-β1 (15 ng/ml) for the indicated time intervals. The conditioned media were collected and analyzed MMP-9 activity by gelatin zymography (upper panel). For MMP-9 protein level, conditioned media were immunoprecipitated with an anti-MMP-9 antibody and analyzed by western blot (lower panel). (B) Cells were pretreated with SB431542 (SB43, 10 μM), NAC (100 μM), U0126 (10 μM), SB202190 (SB20, 10 μM), SP600125 (SP, 10 μM), or Bay11-7082 (Bay, 1 μM) for 1 h, and then treated with TGF-β1 for 24 h. (C) Schematic pathway for TGF-β1-induced ROS-dependent MMP-9 expression and cell migration in RBA-1 cells. Each solid line and arrow represents a step in an activating pathway. TGF-β1 induces MMP-9 expression via TGF-β receptor, ROS-dependent activation of ERK1/2 and JNK1/2, and transcription factor NF-κB pathway, which results in the promotion of cell migration in RBA-1 cells.

Finally, using rat primary cultured astrocytes, we also demonstrated that TGF-β1 induces MMP-9 expression in a time-dependent manner (Figure 8A, upper panel). The condition media were immunoprecipitated with an anti-MMP-9 antibody and analyzed by western blot. As shown in Figure 8A, TGF-β1 induced expression of MMP-9 protein, but not MMP-2 protein, and release into medium (lower panel), indicating that TGF-β1 also induces MMP-9 protein expression and activation in rat primary cultured astrocytes. In addition, pretreatment of rat primary cultured astrocytes with various inhibitors used in RBA-1 cells also significant attenuated TGF-β1-induced MMP-9 expression (Figure 8B). These data demonstrate that, as in RBA-1 cells, TGF-β1-induced MMP-9 expression is also mediated through the same signaling pathways in rat primary culture astrocytes.

## Discussion

MMPs contribute to a wide range of biological activities in several CNS diseases, such as stroke, Alzheimer's disease, and malignant glioma [3]. Among MMPs, MMP-9 expression and activation have been shown to be predominantly elevated by various brain injuries [4,6], suggesting that MMP-9 may be a critical molecule in the degradation of ECM and in the pathophysiology of many brain diseases. Another gelatinase, gelatinase A (MMP-2, 72 kDa), is constitutively expressed and its expression is usually not inducible in several cell types including brain cells. Moreover, TGF-β and related peptides are simultaneously produced and released following injury to the human CNS [48,57]. Despite an obviously important role of TGF-β in brain trauma and diseases, the processes by which TGF-β is implicated in astrocytic functions are not completely understood. A well-established rat astroglial cell line (RBA-1) is derived from dissociated cultures of normal neonatal rat brain tissues [41]. According to various analyses in previous studies, the properties of RBA-1 cells are similar to those of normal astrocytes [41]. Thus, we used a culture model of RBA-1 cells to investigate the mechanisms underlying TGF-β1-induced MMP-9 expression and cellular functional responses. These results suggest that in RBA-1 cells, activation of ROS-dependent ERK1/2 and JNK1/2 linking to NF-κB, mediated through a TGF-β receptor, is essential for TGF-β1-induced MMP-9 gene expression and cell migration. However, previous studies have demonstrated that MMP-2 can be up-regulated by some stimuli such as TGF-β, but usually participates in development of cancer including growth, invasion, and metastasis [25,58].

Abnormal regulation of MAPKs might be implicated in several CNS disorders [8]. Moreover, TGF-β1 has been reported to act as a multifunctional factor through activation of MAPK cascades in different cell types [19,25,34]. In the present study, we found that ERK1/2 and JNK1/2 are required for MMP-9 expression, since RBA-1 cells transfected with dominant negative ERK1 (ΔERK1), ERK2 (ΔERK2) or JNK (ΔJNK) plasmid led to down-regulation of MMP-9 (Figures 3E and 4E). These results are consistent with the MMP-9 expression and secretion through ERK1/2 in rat cortical astrocytes [8,40,59] and the induction of MMP-9 by oxidized low-density lipoprotein via ERK1/2 and JNK1/2 pathways in RBA-1 cells [10]. Our results are consistent with MMP-9 expression through ERK1/2 in transformed keratinocytes [60]. Previously, many reports have indicated that long-term activation of MAPKs may participate in regulating some cellular functions such as gene expression and cell survival [61,62]. Consistent with these reports, our data show that TGF-β1 stimulated JNK1/2 phosphorylation with a maximal response observed within 4 h (Figure 4D), suggesting that long-term phosphorylation of JNK1/2 by TGF-β1 may play a sustained role in up-regulation of MMP-9 in RBA-1 cells. Moreover, we have also demonstrated that either p38 MAPK inhibitor SB202190 or dominant negative mutant (Δp38) have no effect on TGF-β1-induced MMP-9 expression (Figures 4A, C, and 4E). However, recent reports have also indicated that TGF-β-induced MMP-9 expression is mediated through activation of p38 MAPK, but not ERK1/2, in MCF10A human breast epithelial cells [63] and in human glioblastoma cells [64]. The different results may be due to diverse cell types and experimental conditions.

ROS have been shown to exert a key role in the physiological functions and pathological processes [65-67]. In the brain, ROS also extend to the control of vascular tone which is tightly modulated by metabolic activity within neurons [68]. Moreover, increasing oxidative stress (*i.e*. ROS production) by diverse stimuli can regulate the expression of inflammatory genes linked to pathogenesis of human CNS disorders [65-69]. Recently, increasing evidence attributes the cellular damage in neurodegenerative disorders such as AD to oxidative stress that is due to generation of free radicals implicated in brain inflammatory disorders [30,32]. The effects of TGF-β on ROS generation have been reported to be involved in pathogenesis of tumor progression, connective tissue degradation, and lung disease [33,34,70]. In this study, we found that TGF-β1-induced MMP-9 expression is mediated through ROS generation, since pretreatment with ROS scavenger NAC significantly attenuated TGF-β1-induced responses (Figures 5A-C). The role of ROS in TGF-β1-induced ERK1/2 and JNK1/2 phosphorylation was further confirmed by pretreatment with NAC (Figure 5D), suggesting that ROS-dependent activation of ERK1/2 and JNK1/2 is involved in TGF-β1-induced MMP-9 expression in RBA-1 cells. Consistently, many reports have also shown that MAPKs are the down-stream signaling molecules regulated by ROS [34,70]. In addition, we demonstrated that ROS participates in up-regulation of MMP-9 by direct exposure of RBA-1 cells to H_2_O_2 _(Figure 5F). Herein we are the first to establish that intracellular ROS generation contributes to up-regulation of MMP-9 induced by TGF-β1 in RBA-1 cells.

NF-κB is a well-known redox-regulated transcription factor for expression of genes induced by diverse stress signals, including mutagenic, oxidative, and hypoxic stresses associated with physiological and pathological events. Our results reveal that TGF-β1-induced MMP-9 expression via NF-κB phosphorylation, is mediated through ROS-dependent ERK1/2 and JNK1/2 cascades in RBA-1 cells (Figure 6). The requirement of NF-κB signaling for MMP-9 induction has been confirmed by *in vitro *and *in vivo *studies [40,53], which demonstrate a relationship between MMP-9 expression and enhancing cell motility [9,10] and tumor invasion [53]. In RBA-1 cells and human U87 astrocytoma cells, ERK1/2 has been suggested to be necessary for NF-κB activation [40,71]. In addition, accumulating evidence also indicates that TGF-β1-triggered urokinase up-regulation and promotion of invasion is mediated through an ERK1/2-dependent, but not p38 MAPK, activation of NF-κB in human ovarian cancer cells [72]. Our previous study of RBA-1 cells has indicated that up-regulation of MMP-9 by BK is mediated through an ERK1/2-dependent NF-κB pathway [40]. Recently, the JNK/NF-κB cascade has also been shown to participate in TGF-β1-induced MMP-9 expression in corneal epithelial cells [36]. These data imply that different MAPK members are differentially involved in NF-κB activation in various cell types. These studies are consistent with our presented results in RBA-1 cells challenged with TGF-β1.

Cell migration is essential for the organization and maintenance of tissue integrity and plays a role in embryonic development, wound healing, inflammation, and invasiveness through ECM [73,74]. It has been reported that ROS, MAPKs, and NF-κB are involved in MMP-9 up-regulation, which is crucial for regulating cell motility in different cell types [9,56,75,76]. In this study, we demonstrated that TGF-β1-enhanced cell migration is mediated through up-regulation of MMP-9 protein and activity (Figure 1E) via TGF-β receptor and ROS-dependent NF-κB cascade (Figures 2C, 5E, and 6E). Moreover, to rule out the possibility of cell proliferation in TGF-β1-induced cell migration, hydroxyurea, an inhibitor of DNA synthesis [77], was used to prevent proliferation of astrocytes during the period of observation in the migration (wound healing) assay. Therefore, these results suggest that up-regulation of MMP-9 by TGF-β1 is essential for enhancing migration of RBA-1 cells.

## Conclusion

In the study, we have demonstrated that TGF-β1 directly induces MMP-9 expression via TGF-β receptor, ROS-dependent activation of ERK1/2 and JNK1/2, and transcription factor NF-κB pathway, which results in the promotion of cell migration in RBA-1 cells. Based on observations from the literature and on our findings, Figure 8C depicts a model for the molecular mechanisms underlying TGF-β1-induced MMP-9 expression and migration of RBA-1 cells. These findings imply that TGF-β1 might play a critical role in the processes of wound healing and scar formation after brain injuries and diseases. Pharmacological approaches suggest that targeting MMP-9 and their upstream signaling components may yield useful therapeutic targets for the treatment of brain injury, tumors, and inflammatory diseases.

## Competing interests

The authors declare that they have no competing interests.

## Authors' contributions

HLH designed experiments, co-conceived of the study, and drafted the manuscript. HHW, WBW, and PJC designed and performed experiments, and co-conceived of the study. CMY co-conceived of the study, participated in its design and coordination, has been involved in drafting the manuscript and revising it critically for important intellectual content and has given final approval of the version to be published. All authors have read and approved the final version of this manuscript.

## References

[B1] Dollery CM McEwan JR Henney AM Matrix metalloproteinases and cardiovascular disease Circ Res 1995 77 863 868 7554139 10.1161/01.res.77.5.863

[B2] Yong VW Krekoski CA Forsyth PA Bell R Edwards DR Matrix metalloproteinases and diseases of the CNS Trends Neurosci 1998 21 75 80 10.1016/S0166-2236(97)01169-7 9498303

[B3] Yong VW Power C Forsyth P Edwards DR Metalloproteinases in biology and pathology of the nervous system Nat Rev Neurosci 2001 2 502 511 10.1038/35081571 11433375 PMC7097548

[B4] Aoki T Sumii T Mori T Wang X Lo EH Blood-brain barrier disruption and matrix metalloproteinase-9 expression during reperfusion injury: mechanical versus embolic focal ischemia in spontaneously hypertensive rats Stroke 2002 33 2711 2717 10.1161/01.STR.0000033932.34467.97 12411666

[B5] Harris JE Nuttall RK Elkington PT Green JA Horncastle DE Graeber MB Edwards DR Friedland JS Monocyte-astrocyte networks regulate matrix metalloproteinase gene expression and secretion in central nervous system tuberculosis in vitro and in vivo J Immunol 2007 178 1199 1207 17202385 10.4049/jimmunol.178.2.1199

[B6] Wang X Mori T Jung JC Fini ME Lo EH Secretion of matrix metalloproteinase-2 and -9 after mechanical trauma injury in rat cortical cultures and involvement of MAP kinase J Neurotrauma 2002 19 615 625 10.1089/089771502753754082 12042096

[B7] Gottschall PE Yu X Cytokines regulate gelatinase A and B (matrix metalloproteinase 2 and 9) activity in cultured rat astrocytes J Neurochem 1995 64 1513 1520 10.1046/j.1471-4159.1995.64041513.x 7891077

[B8] Lee WJ Shin CY Yoo BK Ryu JR Choi EY Cheong JH Ryu JH Ko KH Induction of matrix metalloproteinase-9 (MMP-9) in lipopolysaccharide-stimulated primary astrocytes is mediated by extracellular signal-regulated protein kinase 1/2 (Erk1/2) Glia 2003 41 15 24 10.1002/glia.10131 12465042

[B9] Hsieh HL Wu CY Yang CM Bradykinin induces matrix metalloproteinase-9 expression and cell migration through a PKC-δ-dependent ERK/Elk-1 pathway in astrocytes Glia 2008 56 619 632 10.1002/glia.20637 18240315

[B10] Wang HH Hsieh HL Wu CY Sun CC Yang CM Oxidized low-density lipoprotein induces matrix metalloproteinase-9 expression via a p42/p44 and JNK-dependent AP-1 pathway in brain astrocytes Glia 2009 57 24 38 10.1002/glia.20732 18661553

[B11] Flanders KC Ren RF Lippa CF Transforming growth factor-βs in neurodegenerative disease Prog Neurobiol 1998 54 71 85 10.1016/S0301-0082(97)00066-X 9460794

[B12] Massagué J How cells read TGF-β signals Nat Rev Mol Cell Biol 2000 1 169 178 11252892 10.1038/35043051

[B13] Böttner M Krieglstein K Unsicker K The transforming growth factor-βs: structure, signaling, and roles in nervous system development and functions J Neurochem 2000 75 2227 2240 11080174 10.1046/j.1471-4159.2000.0752227.x

[B14] Wynn TA Cellular and molecular mechanisms of fibrosis J Pathol 2008 214 199 210 10.1002/path.2277 18161745 PMC2693329

[B15] Vivien D Ali C Transforming growth factor-β signalling in brain disorders Cytokine Growth Factor Rev 2006 17 121 128 10.1016/j.cytogfr.2005.09.011 16271500

[B16] Wakefield LM Roberts AB TGF-β signaling: positive and negative effects on tumorigenesis Curr Opin Genet Dev 2002 12 22 29 10.1016/S0959-437X(01)00259-3 11790550

[B17] Bierie B Moses HL TGF-β and cancer Cytokine Growth Factor Rev 2006 17 29 40 10.1016/j.cytogfr.2005.09.006 16289860

[B18] Verrecchia F Mauviel A Farge D Transforming growth factor-β signaling through the Smad proteins: role in systemic sclerosis Autoimmun Rev 2005 5 563 569 10.1016/j.autrev.2006.06.001 17027893

[B19] Gomes FC Sousa Vde O Romão L Emerging roles for TGF-β1 in nervous system development Int J Dev Neurosci 2005 23 413 424 10.1016/j.ijdevneu.2005.04.001 15936920

[B20] Lehrmann E Kiefer R Christensen T Toyka KV Zimmer J Diemer NH Hartung HP Finsen B Microglia and macrophages are major sources of locally produced transforming growth factor-β1 after transient middle cerebral artery occlusion in rats Glia 1998 24 437 448 10.1002/(SICI)1098-1136(199812)24:4<437::AID-GLIA9>3.0.CO;2-X 9814824

[B21] Ruocco A Nicole O Docagne F Ali C Chazalviel L Komesli S Yablonsky F Roussel S MacKenzie ET Vivien D Buisson A A transforming growth factor-β antagonist unmasks the neuroprotective role of this endogenous cytokine in excitotoxic and ischemic brain injury J Cereb Blood Flow Metab 1999 19 1345 1353 10.1097/00004647-199912000-00008 10598939

[B22] Pratt BM McPherson JM TGF-β in the central nervous system: potential roles in ischemic injury and neurodegenerative diseases Cytokine Growth Factor Rev 1997 8 267 292 10.1016/S1359-6101(97)00018-X 9620642

[B23] Zhu Y Yang GY Ahlemeyer B Pang L Che XM Culmsee C Klumpp S Krieglstein J Transforming growth factor-β1 increases bad phosphorylation and protects neurons against damage J Neurosci 2002 22 3898 3909 12019309 10.1523/JNEUROSCI.22-10-03898.2002 PMC6757635

[B24] ten Dijke P Hill CS New insights into TGF-β-Smad signalling Trends Biochem Sci 2004 29 265 273 10.1016/j.tibs.2004.03.008 15130563

[B25] Leivonen SK Kähäri VM Transforming growth factor-β signaling in cancer invasion and metastasis Int J Cancer 2007 121 2119 2124 10.1002/ijc.23113 17849476

[B26] Massagué J Wotton D Transcriptional control by the TGF-β/Smad signaling system EMBO J 2000 19 1745 1754 10775259 10.1093/emboj/19.8.1745 PMC302010

[B27] Buisson A Lesne S Docagne F Ali C Nicole O MacKenzie ET Vivien D Transforming growth factor-β and ischemic brain injury Cell Mol Neurobiol 2003 23 539 550 10.1023/A:1025072013107 14514014 PMC11530161

[B28] Leivonen SK Chantry A Hakkinen L Han J Kahari VM Smad3 mediates transforming growth factor-β-induced collagenase-3 (matrix metalloproteinase-13) expression in human gingival fibroblasts. Evidence for cross-talk between Smad3 and p38 signaling pathways J Biol Chem 2002 277 46338 46346 10.1074/jbc.M206535200 12270924

[B29] Leivonen SK Häkkinen L Liu D Kähäri VM Smad3 and extracellular signal-regulated kinase 1/2 coordinately mediate transforming growth factor-β-induced expression of connective tissue growth factor in human fibroblasts J Invest Dermatol 2005 24 1162 1169 10.1111/j.0022-202X.2005.23750.x 15955090

[B30] Lewén A Matz P and Chan PH Free radical pathways in CNS injury J Neurotrauma 2000 17 871 890 11063054 10.1089/neu.2000.17.871

[B31] Chan PH Reactive oxygen radicals in signaling and damage in the ischemic brain J Cereb Blood Flow Metab 2001 21 2 14 10.1097/00004647-200101000-00002 11149664

[B32] Halliwell B Oxidative stress and neurodegeneration: where are we now? J Neurochem 2006 97 1634 1658 10.1111/j.1471-4159.2006.03907.x 16805774

[B33] Liu RM Oxidative stress, plasminogen activator inhibitor 1, and lung fibrosis Antioxid Redox Signal 2008 10 303 319 10.1089/ars.2008.2121 17979497 PMC3686819

[B34] Koli K Myllärniemi M Keski-Oja J Kinnula VL Transforming growth factor-β activation in the lung: focus on fibrosis and reactive oxygen species Antioxid Redox Signal 2008 10 333 342 10.1089/ars.2007.1914 17961070

[B35] Han YP Tuan TL Hughes M Wu H Garner WL Transforming growth factor-β-and tumor necrosis factor-α-mediated induction and proteolytic activation of MMP-9 in human skin J Biol Chem 2001 276 22341 22350 10.1074/jbc.M010839200 11297541 PMC2651823

[B36] Gordon GM Ledee DR Feuer WJ Fini ME Cytokines and signaling pathways regulating matrix metalloproteinase-9 (MMP-9) expression in corneal epithelial cells J Cell Physiol 2009 221 402 411 10.1002/jcp.21869 19626678 PMC2990951

[B37] Rosenberg GA Matrix metalloproteinases in neuroinflammation Glia 2002 39 279 291 10.1002/glia.10108 12203394

[B38] Sato H Kita M Seiki M v-Src activates the expression of 92-kDa type IV collagenase gene through the AP-1 site and the GT box homologous to retinoblastoma control elements. A mechanism regulating gene expression independent of that by inflammatory cytokines J Biol Chem 1993 268 23460 23468 8226872

[B39] Wu CY Hsieh HL Jou MJ Yang CM Involvement of p42/p44 MAPK, p38 MAPK, JNK and nuclear factor-κB in interleukin-1β-induced matrix metalloproteinase-9 expression in rat brain astrocytes J Neurochem 2004 90 1477 1488 10.1111/j.1471-4159.2004.02682.x 15341531

[B40] Hsieh HL Yen MH Jou MJ Yang CM Intracellular signalings underlying bradykinin-induced matrix metalloproteinase-9 expression in rat brain astrocyte-1 Cell Signal 2004 16 1163 1176 10.1016/j.cellsig.2004.03.021 15240011

[B41] Jou TC Jou MJ Chen JY Lee SY Properties of rat brain astrocytes in long-term culture J Formos Med Assoc 1985 84 865 881 3865993

[B42] LeBel CP Ischiropoulos H Bondy SC Evaluation of the probe 2',7'-dichlorofluorescein as an indicator of reactive oxygen species formation and oxidative stress Chem Res Toxicol 1992 5 227 231 10.1021/tx00026a012 1322737

[B43] Eberhardt W Schulze M Engels C Klasmeier E Pfeilschifter J Glucocorticoid-mediated suppression of cytokine-induced matrix metalloproteinase-9 expression in rat mesangial cells: involvement of nuclear factor-κB and Ets transcription factors Mol Endocrinol 2002 16 1752 1766 10.1210/me.2001-0278 12145332

[B44] Obrig TG Culp WJ McKeehan WL Hardesty B The mechanism by which cycloheximide and related glutarimide antibiotics inhibit peptide synthesis on reticulocyte ribosomes J Biol Chem 1971 246 174 181 5541758

[B45] Inman GJ Nicolas FJ Callahan JF Harling JD Gaster LM Reith AD Laping NJ Hill CS SB-431542 is a potent and specific inhibitor of transforming growth factor-beta superfamily type I activin receptor-like kinase (ALK) receptors ALK4, ALK5, and ALK7 Mol Pharmacol 2002 62 65 74 10.1124/mol.62.1.65 12065756

[B46] Laping NJ Grygielko E Mathur A Butter S Bomberger J Tweed C Martin W Fornwald J Lehr R Harling J Gaster L Callahan JF Olson BA Inhibition of transforming growth factor (TGF)-β1-induced extracellular matrix with a novel inhibitor of the TGF-β type I receptor kinase activity: SB-431542 Mol Pharmacol 2002 62 58 64 10.1124/mol.62.1.58 12065755

[B47] Jeon ES Moon HJ Lee MJ Song HY Kim YM Bae YC Jung JS Kim JH Sphingosylphosphorylcholine induces differentiation of human mesenchymal stem cells into smooth-muscle-like cells through a TGF-β-dependent mechanism J Cell Sci 2006 119 4994 5005 10.1242/jcs.03281 17105765

[B48] Dhandapani KM Brann DW Transforming growth factor-β: a neuroprotective factor in cerebral ischemia Cell Biochem Biophys 2003 39 13 22 10.1385/CBB:39:1:13 12835526

[B49] Powrozek TA Miller MW Ethanol affects transforming growth factor β1-initiated signals: cross-talking pathways in the developing rat cerebral wall J Neurosci 2009 29 9521 9533 10.1523/JNEUROSCI.2371-09.2009 19641115 PMC6666549

[B50] Moon SK Kang SK Kim CH Reactive oxygen species mediates disialoganglioside GD3-induced inhibition of ERK1/2 and matrix metalloproteinase-9 expression in vascular smooth muscle cells FASEB J 2006 20 1387 1395 10.1096/fj.05-4618com 16816114

[B51] Shin MH Moon YJ Seo JE Lee Y Kim KH Chung JH Reactive oxygen species produced by NADPH oxidase, xanthine oxidase, and mitochondrial electron transport system mediate heat shock-induced MMP-1 and MMP-9 expression Free Radic Biol Med 2008 44 635 645 10.1016/j.freeradbiomed.2007.10.053 18036352

[B52] Hempel N Ye H Abessi B Mian B Melendez JA Altered redox status accompanies progression to metastatic human bladder cancer Free Radic Biol Med 2009 46 42 50 10.1016/j.freeradbiomed.2008.09.020 18930813 PMC2630461

[B53] Tai KY Shieh YS Lee CS Shiah SG Wu CW Axl promotes cell invasion by inducing MMP-9 activity through activation of NF-κB and Brg-1 Oncogene 2008 27 4044 4055 10.1038/onc.2008.57 18345028

[B54] Ghosh S Hayden MS New regulators of NF-κB in inflammation Nat Rev Immunol 2008 8 837 848 10.1038/nri2423 18927578

[B55] Krohn K Rozovsky I Wals P Teter B Anderson CP Finch CE Glial fibrillary acidic protein transcription responses to transforming growth factor-β1 and interleukin-1β are mediated by a nuclear factor-1-like site in the near-upstream promoter J Neurochem 1999 72 1353 1361 10.1046/j.1471-4159.1999.721353.x 10098836

[B56] Huang TT Feinberg SL Suryanarayanan S Miyamoto S The zinc finger domain of NEMO is selectively required for NF-κB activation by UV radiation and topoisomerase inhibitors Mol Cell Biol 2002 22 5813 5825 10.1128/MCB.22.16.5813-5825.2002 12138192 PMC133970

[B57] Bruno V Battaglia G Casabona G Copani A Caciagli F Nicoletti F Neuroprotection by glial metabotropic glutamate receptors is mediated by transforming growth factor-β J Neurosci 1998 18 9594 9600 9822720 10.1523/JNEUROSCI.18-23-09594.1998 PMC6793276

[B58] Kim ES Sohn YW Moon A TGF-beta-induced transcriptional activation of MMP-2 is mediated by activating transcription factor (ATF)2 in human breast epithelial cells Cancer Lett 2007 252 147 156 10.1016/j.canlet.2006.12.016 17258390

[B59] Arai K Lee SR Lo EH Essential role for ERK mitogen-activated protein kinase in matrix metalloproteinase-9 regulation in rat cortical astrocytes Glia 2003 43 254 264 10.1002/glia.10255 12898704

[B60] Santibáñez JF Guerrero J Quintanilla M Fabra A Martínez J Transforming growth factor-β1 modulates matrix metalloproteinase-9 production through the Ras/MAPK signaling pathway in transformed keratinocytes Biochem Biophys Res Commun 2002 296 267 273 12163012 10.1016/s0006-291x(02)00864-1

[B61] Wang WH Grégori G Hullinger RL Andrisani OM Sustained Activation of p38 Mitogen-Activated Protein Kinase and c-Jun N-Terminal Kinase Pathways by Hepatitis B Virus × Protein Mediates Apoptosis via Induction of Fas/FasL and Tumor Necrosis Factor (TNF) Receptor 1/TNF-α Expression Mol Cell Biol 2004 24 10352 10365 10.1128/MCB.24.23.10352-10365.2004 15542843 PMC529056

[B62] Seok JH Park KA Byun HS Won M Shin S Choi BL Lee H Kim YR Hong JH Park J Hur GM Long-term Activation of c-Jun N-terminal Kinase through Receptor Interacting Protein is Associated with DNA Damage-induced Cell Death Korean J Physiol Pharmacol 2008 12 185 191 10.4196/kjpp.2008.12.4.185 19967054 PMC2788634

[B63] Kim ES Kim MS Moon A TGF-β-induced upregulation of MMP-2 and MMP-9 depends on p38 MAPK, but not ERK signaling in MCF10A human breast epithelial cells Int J Oncol 2004 25 1375 1382 15492828

[B64] Dziembowska M Danilkiewicz M Wesolowska A Zupanska A Chouaib S Kaminska B Cross-talk between Smad and p38 MAPK signalling in transforming growth factor beta signal transduction in human glioblastoma cells Biochem Biophys Res Commun 2007 354 1101 1106 10.1016/j.bbrc.2007.01.113 17276399

[B65] Floyd RA Neuroinflammatory processes are important in neurodegenerative diseases: an hypothesis to explain the increased formation of reactive oxygen and nitrogen species as major factors involved in neurodegenerative disease development Free Radic Biol Med 1999 26 1346 1355 10.1016/S0891-5849(98)00293-7 10381209

[B66] Kamata H Hirata H Redox regulation of cellular signalling Cell Signal 1999 11 1 14 10.1016/S0898-6568(98)00037-0 10206339

[B67] Valko M Leibfritz D Moncol J Cronin MT Mazur M Telser J Free radicals and antioxidants in normal physiological functions and human disease Int J Biochem Cell Biol 2007 39 44 84 10.1016/j.biocel.2006.07.001 16978905

[B68] Demchenko IT Oury TD Crapo JD Piantadosi CA Regulation of the brain's vascular responses to oxygen Circ Res 2002 91 1031 1037 10.1161/01.RES.0000043500.03647.81 12456489

[B69] Infanger DW Sharma RV Davisson RL NADPH oxidases of the brain: distribution, regulation, and function Antioxid Redox Signal 2006 8 1583 1596 10.1089/ars.2006.8.1583 16987013

[B70] Wu WS The signaling mechanism of ROS in tumor progression Cancer Metastasis Rev 2006 25 695 705 10.1007/s10555-006-9037-8 17160708

[B71] Kam AY Liu AM Wong YH Formyl peptide-receptor like-1 requires lipid raft and extracellular signal-regulated protein kinase to activate inhibitor-κB kinase in human U87 astrocytoma cells J Neurochem 2007 103 1553 1566 10.1111/j.1471-4159.2007.04876.x 17727628

[B72] Tanaka Y Kobayashi H Suzuki M Kanayama N Terao T Transforming growth factor-β1-dependent urokinase up-regulation and promotion of invasion are involved in Src-MAPK-dependent signaling in human ovarian cancer cells J Biol Chem 2004 279 8567 8576 10.1074/jbc.M309131200 14676209

[B73] Liotta LA Kohn E Cancer invasion and metastases JAMA 1990 263 1123 1126 10.1001/jama.263.8.1123 2405205

[B74] Lauffenburger DA Horwitz AF Cell migration: a physically integrated molecular process Cell 1996 84 359 369 10.1016/S0092-8674(00)81280-5 8608589

[B75] Mantuano E Inoue G Li X Takahashi K Gaultier A Gonias SL Campana WM The hemopexin domain of matrix metalloproteinase-9 activates cell signaling and promotes migration of schwann cells by binding to low-density lipoprotein receptor-related protein J Neurosci 2008 28 11571 11582 10.1523/JNEUROSCI.3053-08.2008 18987193 PMC3837707

[B76] Shinohara M Adachi Y Mitsushita J Kuwabara M Nagasawa A Harada S Furuta S Zhang Y Seheli K Miyazaki H Kamata T Reactive oxygen generated by NADPH oxidase (NOX) 1 contributes to cell division by regulating matrix metalloprotease-9 production and cell migration J Biol Chem 2010 285 4481 4488 10.1074/jbc.M109.071779 20018867 PMC2836054

[B77] Yarbro JW Mechanism of action of hydroxyurea Semin Oncol 1992 19 1 10 1641648

